# The Gastroscope

**Published:** 1882-02

**Authors:** 


### THE GASTROSCOPE.

Dr. Mikulicz, of Vienna, has invented an instrument for illuminating and inspecting the inside of the living human stomach. On November 5 he exhibited his apparatus, upon which he has bestowed the title of "Gastroscope" to the leading professors of the leading medical faculty at the polyklinik, and performed some interesting experiments with it upon a female hospital patient suffering from chronic dyspepsia. It consists of a tube, fitted with a set of minute but powerful reflectors at one end, and connected at the other with an electric battery, by which a brilliant light is projected into the stomach requiring inspection. This tube was passed down the subject's throat and remained there for fully twenty minutes, during which time the Viennese professors were enabled to diagnose the condition of every part of the mucous membrane thus lighted up and revealed to their gaze. The Gastroscope is considered likely to render invaluable services to the cause of electro-endoscopic investigation, which for some time past has been prosecuted with ardor by eminent Austrian pathologists.

